# Preparation of a cellulose acetate membrane using cigarette butt recycling and investigation of its efficiency in removing heavy metals from aqueous solution

**DOI:** 10.1038/s41598-022-24432-x

**Published:** 2022-11-25

**Authors:** Javad Torkashvand, Alireza Saeedi-Jurkuyeh, Roshanak Rezaei Kalantary, Mitra Gholami, Ali Esrafili, Mahmood Yousefi, Mahdi Farzadkia

**Affiliations:** 1 Research Center for Environmental Health Technology, Iran University of Medical Sciences, Tehran, Iran; 2 Department of Environmental Health Engineering, School of Public Health, Iran University of Medical Sciences, Tehran, Iran; 3 Department of Environmental Health Engineering, Deputy of Health, Guilan University of Medical Sciences, Rasht, Iran

**Keywords:** Environmental sciences, Environmental social sciences, Chemistry

## Abstract

This study investigated the recycling of freshly-smoked cigarette butts (FCBs) and unsmoked cigarette filters (UCFs) into a cellulose acetate (CA) membrane. The both samples were prepared by means of a combination of seven cigarette brands, and the phase inversion method was used to recycle each sample into a membrane using N-methyl-2-pyrrolidone. The efficiency of the prepared membranes for the removal of chromium, cadmium, and lead from an aqueous solution in a forward osmosis reactor was investigated. The results showed that the both membranes had a smooth surface and macrovoids. The flux of the prepared membranes from the UCFs and FCBs recycling were 14.8 and 13.2 LMH, respectively. The porosity and reverse salt of the UCFs membrane were 61% and 3.5 gMH, while those for FCBs membrane were 58% and 3.9 gMH. The observed metal removal efficiency of the both membranes was in the range of 85 to 90%. However, increasing the concentration of metals up to five times caused a slight decrease in the removal efficiency (less than 5%).

## Introduction

When the first filtered cigarettes were introduced in the 1950s to reduce harmful elements inhaled by smokers^1^, it seemed that this type of cigarette could control the health consequences of smoking. Filtered cigarettes have been able to reduce the risk of smoking due to the ability of their filter via trapping harmful contaminants from cigarette smoke^2^, and today they are the most common form of tobacco use worldwide^3^. However, filtered cigarettes have posed a serious environmental risk. Cigarette butts (CBs), which are often littered as waste after smoking, are now recognized as a major environmental polluter contaminating many public places worldwide^4^. More than 4.5 trillion CBs are littered annually^5^, making them one of the most prevalent hazardous wastes across the globe. Furthermore, the number of littered CBs is expected to increase to nearly 2 million tons per year in the world^6^.

In addition to the great number of CBs, their dispersion in the environment is another dangerous aspect of this hazardous waste; also, since many smokers carelessly discard CBs, this waste has been considered one of the most common item of litter in the environment^7–10^. As a consequence, the management of this waste is facing serious practical challenges, including the high costs of littered CBs collection^11^. Moreover, there is no efficient solution for collecting CBs from urban environments and public places such as beaches^12^. Furthermore, CBs are known as a hazardous waste because they contain thousands of chemical components such as heavy metals and toxins. Because these harmful contents often leach into the environment, CBs pose a potential threat to the environment, human health, and local organisms^13^. Chemicals leaching from littered CBs is a serious issue as it leads to soil and water pollution. In fact, leached nicotine from a cigarette butt has the potential to pollute 1000 L of water^14^. Besides, CB leachate is toxic to plants and animals. The literature reviews have revealed that CBs can significantly reduce plant growth and change the normal size of organs in some animals^15,16^. Another environmental threat related to littered CBs is the risk of ingestion by domestic animals and wildlife^17,18^.

Therefore, it is essential to seek efficient solutions to tackle this environmental issue. However, another challenge associated with CB management is the limitation over the use of conventional waste disposal methods such as landfilling and incineration. The both mentioned techniques may result in the release of hazardous chemicals into the air, water, and soil and are not suggested as a proper measure to manage CBs^19^. However, numerous studies with encouraging findings have been published on recycling CBs in recent years, such as extraction of trapped chemicals in CBs for vector control^20,21^. Numerous attempts have been made in this field, for instance, the production of biofilm carriers used in wastewater treatment^22^, carbon adsorbents^23,24^, bricks and asphalt^25,26^, sound absorbers^27^, and paper pulp^28^ from CBs have been investigated in previous studies.

In the last decade, numerous attempts have been made to recycle CBs into various products and extract the trapped chemicals in their filter for different purposes. Cigarette butt recycling goals can be categorized into three main groups.

1. The use of trapped chemicals in the filter for purposes like vector control^20^ and metal corrosion control^29^.
2. Converting the recovered cellulose acetate from CBs into valuable products such as paper pulp^28^ and super-capacitor^30^.
3. The use of whole CBs without separating their components for brick and similar product manufacturing^25^.

Products with good quality and performance will ensure sustainable recycling of CBs as a hazardous waste and an environmental challenge. However, in many previous attempts in this field, the quality of the final product was not satisfying compared to commercial samples. The produced bricks using the addition of CBs to the raw materials did not have the same thermal features like the heat resistance and heat transfer as commercial samples^25^.

The paper converted from CB recycling in the study conducted by Teixeira et al. looked darker and was more brittle than commercial paper^28^. Furthermore, in various studies on CB recycling to carbon adsorbents, the adsorption capacity in recycled samples has been reported to be moderate compared to commercial adsorbents^23,24^. However, the quality of some other final products from CB recycling has reported to be satisfying. Ou et al. successfully recycled CBs into oileophilic fibers, which remained efficient after 10 experimental runs^31^. The biofilm carrier prepared from CB recycling in the study by Sabzali et al. showed similar performance as commercial samples used in wastewater treatment^22^.

However, there are significant challenges facing CBs recycling including the threat of contaminants leakage during the process and the quality of the final product^19^. Considering that nearly 2 million tons of CB waste is produced each year globally, one of the most important challenges in CB recycling is selecting high-demand products as CB recycling output. Given that cigarette filters are made from cellulose acetate fibers^4,5^, the production of cellulose acetate based products can be a good solution for CB recycling on a large scale. Membranes have numerous applications in today’s world, such as water and wastewater treatment^32–35^, and cellulose acetate is one of the most common materials used for membrane production^36–38^, offering a great opportunity to manage CBs by recycling them into membranes. This study aimed to investigate the possibility of CB recycling into a membrane and the characteristics of the produced membrane. Furthermore, the efficiency of the produced membrane in the removal of chromium, cadmium, and lead from an aqueous solution in a forward osmosis reactor was examined and compared with other available membranes.

## Materials and methods

### Preparation of cigarette butt samples

Two samples including freshly smoked cigarette butts (FCBs) and unsmoked cigarette filters (UCFs) were prepared. In order to prepare UCFs sample, seven best-selling cigarette brands were identified in Iran’s market. Three pockets (60 filtered cigarettes) were bought from each brand in three different sales centers. The filters were separated and mixed and then used without processing. The FCB sample was prepared from the same brands using a hand pump with ten suctions for each cigarette. The remaining tobacco from obtained CBs was removed in the initial stage of processing. The wrapping papers around the filters were then separated manually. After initial processing, the cleaning process was done by 20-min immersion and mixing in water three times^19,23,24^. The filters were then immersed in 96% ethanol twice for 20 min. Finally, an acidic solution containing nitric acid and acetic acid was used to remove heavy metals^39^. The cleaned filters were kept at room temperature for 48 h to dry completely before entering the main recycling stage.

### Membrane preparation

**Figure 1:**
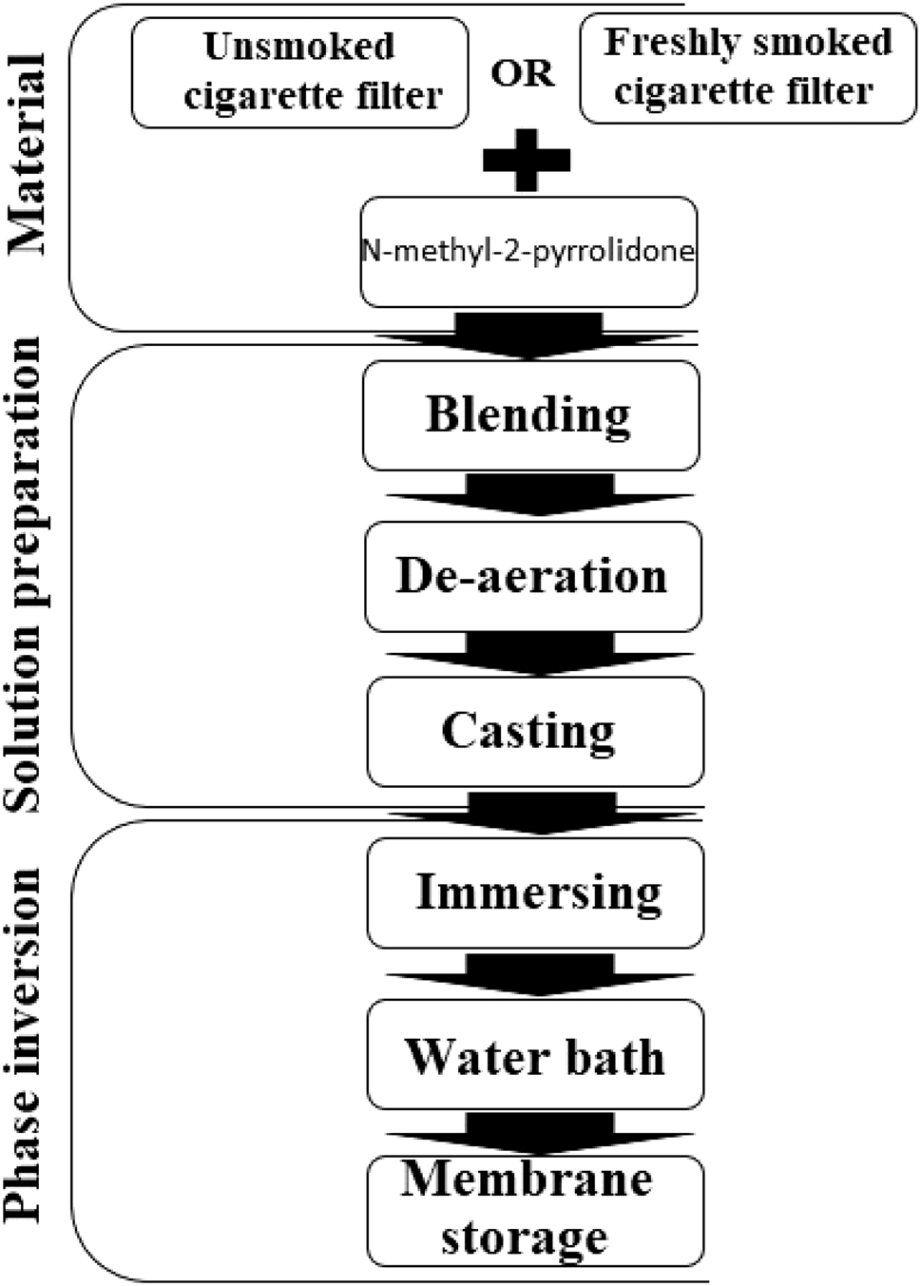
Membrane preparation steps.

Membrane preparation was performed based on the phase inversion method^40,41^. Considering the effect of viscosity on miscibility, based on previous studies; for each membrane preparation, the filters were added to N-methyl-2-pyrrolidone solvent at a ratio of 15 wt%^38,41^. Due to the existence of many previous studies in the field of membrane preparation from cellulose acetate, in this study, a cellulose acetate-based membrane was not prepared. Thus, the performance of the prepared membrane by UCF and FSB were compared with the results of previous studies. To achieve a homogeneous solution, the mixture was placed in a magnetic stirrer for 8 h to a temperature of 40 °C. The generated air bubbles must be removed from the mixture before casting, so the mixture was kept at room temperature for 2 h and then transferred to the refrigerator and kept at 4 °C for 24 hours^36,42^. The degassed solution was cast, and a thin film with a thickness of 150 μm was formed. The yielded film was immediately immersed in deionized water for 15 min to undergo the phase inversion^41,42^. The film was placed in a 50 °C water bath for 15 min to complete phase inversion and solvent replacement with water^42,43^. The resulting membrane was kept in distilled water at room temperature for 48 h to ensure complete removal of the solvent and impurities^43^. Finally, the prepared membrane was kept in distilled water at 4 °C before morphology and characteristics evaluation^40^. Membrane preparation steps are shown in Fig. 1.

### Membrane properties

**Figure 2:**
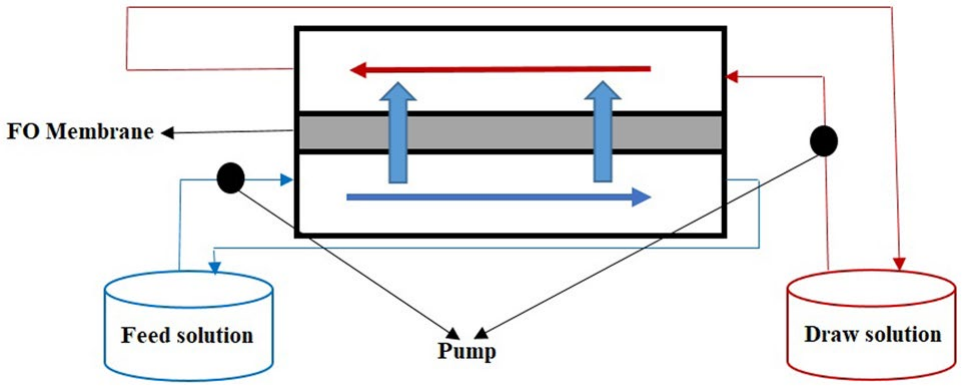
Schematic of the used forward osmosis system.

The morphology and porosity structure of the prepared membranes were studied using the surface and cross-section images taken by a scanning electron microscope (SEM) according to the presented method in previous studies^36,41,42^. The membranes were frozen in liquid nitrogen for 60 s, and then the frozen fragments were broken and coated with gold by sputtering technique to produce electric conductivity^42^. A laboratory-scale forward osmosis system was utilized to evaluate the operational characteristics of the prepared membranes and their efficiency in heavy metal removal from an aqueous solution. As shown in Fig. 2, this system consist of draw and feed solutions, which are flowed separately by two different pumps. The membrane holding chamber had dimensions of 2.1 cm in width and 3.2 cm in length, which was properly covered the surface area of the membrane equal to 6.7 cm^2^.

In order to determine the water flux of the prepared membrane, forward osmosis was used with (100 ml of 1 M sodium chloride solution) as the draw solution and 400 ml of deionized water as the feed solution. The draw solution was circulated in the system with a flow rate of 18 L per hour, and the membrane flux was calculated using the following equation.

1 $$Jw = \frac{\Delta m}{{\Delta t}} \frac{1}{Am}$$

where JW is the water flux in L/m^2^.h, Δm is the decrease in weight of the feed solution in liters, Am is the active area of the membrane in m^2^, and Δt represents time in hour, which was 0.25 in this study.

Also, to determine the reverse salt flux the following formula was used.

2 $$Js = \frac{\Delta CtV}{{\Delta t}} \frac{1}{Am}$$

where Js is reverse salt flux in g/MH, ΔCt is the salt loss of draw solution in gram, V stands for the decrease in weight of the draw solution in gram, Am represents the active area of the membrane in m^2^, and Δt is time in hour, which was 0.25 in this study.

To determine porosity, wet membrane mass (W1) and dry membrane mass (W2) were measured and placed in the following formula.

3 $${\mathcal{E}} = \frac{{\frac{{w_{1} - w_{2} }}{{\rho_{w} }}}}{{\left( {\frac{{w_{1} - w_{2} }}{{\rho_{w} }}} \right) + \left( {\frac{{w_{2} }}{{\rho_{m} }}} \right)}}100$$

In this equation $\rho_{w}$ and $\rho_{m}$ represent the density of water and the membrane, respectively.

### Investigation the membrane efficiency in metal removal

The efficiency of the prepared membranes in the removal of chromium, cadmium, and lead from an aqueous solution was investigated in the forward osmosis process. The feed solutions for each mentioned heavy metal were prepared in 10, 20, 30, and 50 mg/L concentrations. Furthermore, 1 molar (M) solution of sodium chloride was used as the draw solution. All steps associated with the evaluation of the membrane operational characteristics and its efficiency in heavy metals removal were performed at room temperature. Both flows (draw and feed) were adjusted at 0.3 L per minute, and sampling was performed 20 min after the beginning of the process. The concentration of heavy metals in the solution was measured using the GF-AAS system, made in Australia. The following formula was used to determine the removal efficiency of heavy metals.

4 $${\text{Removal efficiency}} = \frac{C0 - Ce}{{C0}} 100$$

In this equation, $C0$ and $Ce$ represent the concentration of metal in feed solution and draw solution, respectively.

## Results and discussion

### Membrane properties

The morphology of the membrane has a great impact on its efficiency in specific applications; therefore, achieving a proper physical structure is essential during membrane preparation. Morphology of the developed UCF and FCB membranes was determined using a scanning electron microscope. The obtained images demonstrated that the prepared membranes had macrovoids. Membrane formation through CB recycling can be explained based on the phase inversion process. The cigarette filter, which is the main part of CBs, is mainly composed of cellulose acetate^4,5,18^. N-methyl-2-pyrrolidone and CBs were mixed together as the initial solution, resulting in the formation of a viscous mixture. This viscous mixture was used for casting a film, and then the casted film was immediately immersed in distilled water. Phase inversion, which is the miscibility between water and solvent, occurs at this stage. This phenomenon will lead to the exchange of water and solvent due to diffusional flow^42^. Phase inversion happens because of the low miscibility between cellulose acetate (cigarette filter) and solvent and continues until the end of the demixing process, resulting in solidification^44^.

**Figure 3:**
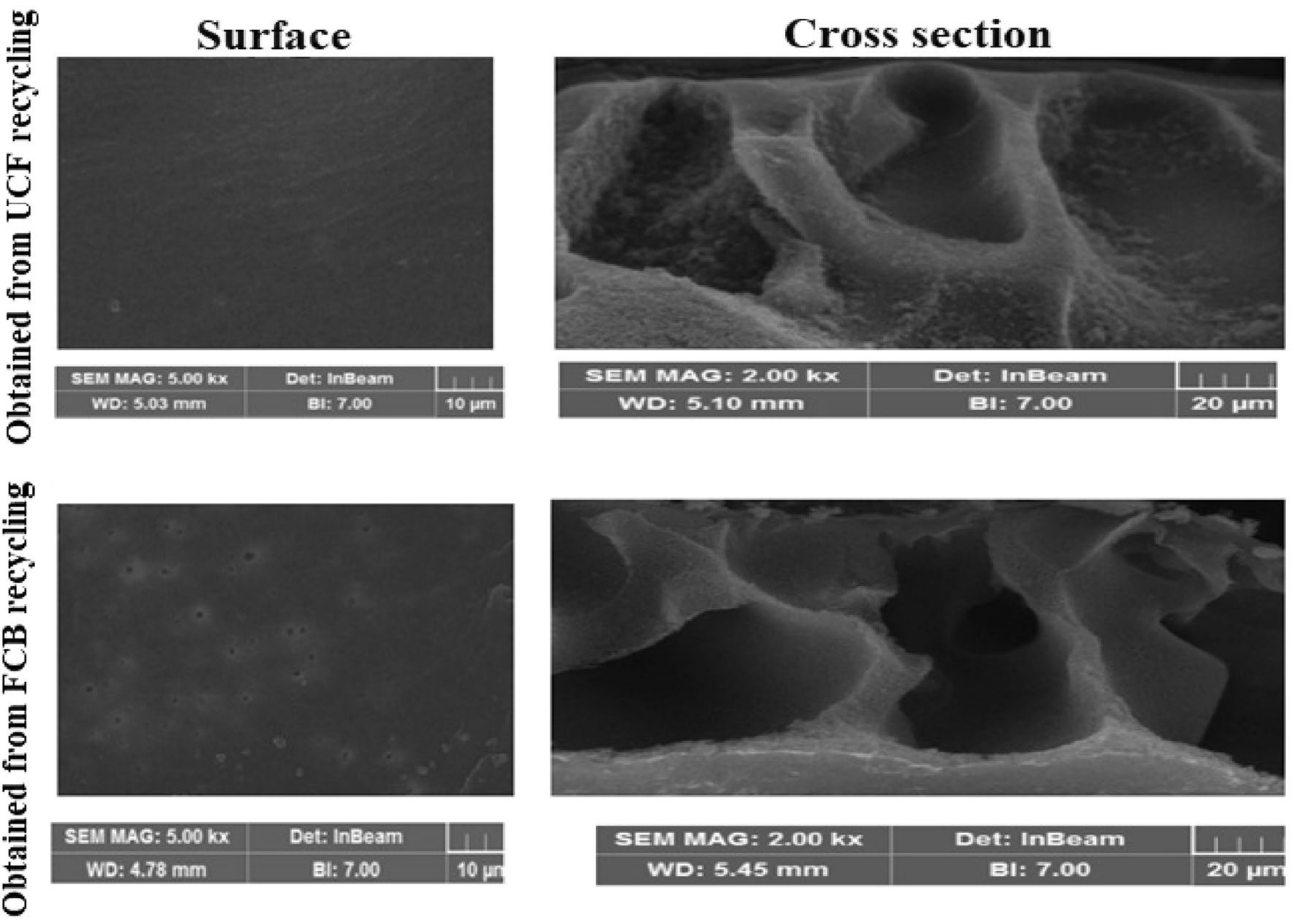
SEM images of the prepared membranes.

As shown in Fig. 3, based on the SEM images of the prepared membranes, both UCF and FCB membranes had a smooth surface. The formation of a smooth surface structure in membranes can be attributed to the speed of the demixing process during phase inversion. In general, membrane morphology depends on the demixing speed, and final membranes with a smooth surface and macrovoids formation indicate instantaneous demixing in the conducted study, whereas if the demixing process was slow, membranes with a dense structure would have formed^42^. Furthermore, as Fig. 3 indicates, there is a slight difference in the number of pores on the surface of the FCB resulting membrane compared to the UCF resulting membrane. This difference may be the effect of cigarette smoke impurities trapped in the filter during the smoking process, which remained in the CBs despite the processing and cleaning stages. The effect of additive compounds on the surface structure of cellulose acetate membranes has been mentioned in similar studies^45,46^. The additive leaching process in the initial mixture preparation and during gelation has been considered a reason for this phenomenon^47^. Accordingly, an increase in the casting solution impurities can lead to a rougher surface of the membrane and more dispersed pores on this surface^42^.

The cross-section SEM images of the prepared membranes from UCF and FCB recycling are shown in Fig. 3. As the figure indicates, there are macrovoids in the membranes. Considering that the morphology of the membrane depends on the thermodynamic conditions and the factors affecting the phase inversion kinetics^48^, the observed structure in the membranes can be explained. The viscosity of casting solution is one of the most important factors affecting the structure of pores and membrane porosity^49,50^. Since the viscosity is effective in the mutual diffusion of solvent and non-solvent, the change in viscosity of the casting solution has a significant impact on the structure of the pores^41^. Due to the fact that the cigarette filters are made from cellulose acetate, the casting solution had low viscosity, leading to suitable porosity and macrovoids in the resulting membranes. In general, a higher viscosity of the casting solution indicates a slower demixing rate in phase inversion, leading to a change in the membrane porosity structure and the formation of a sponge-like structure^36,41^. By contrast, lower viscosity and higher hydrophilicity of the casting solution containing pure cellulose acetate, as used in this study, results in fast phase inversion and, eventually, the formation of a macrovoids and improved porosity in the membrane^42^.

The flux of prepared membranes was measured using deionized water as the feed solution. As Table 1 indicates, the flux of prepared membranes from UCF and FCB recycling were 14.7 and 13.2 L/MH, respectively. Since the cigarette filters are mainly made from cellulose acetate, the low flux observed in resulting membranes can be due to a very tight polymer matrix of pure CA^42^. However, adding some specific compounds to cellulose acetate can increase membrane hydrophilicity and the flux because increased hydrophilicity of the casting solution can raise the flux in the prepared membranes^41,42^. Membrane porosity is another factor that depends on the hydrophilicity of the casting solution and is effective on the flux^51^. As shown in Table 1, the porosity in the prepared membranes from UCT and FCB recycling were 61 and 58%, respectively. Although this amount was close to the porosity of CA membranes^42^, this property can be raised by adding hydrophilic materials to the initial mixture. The addition of hydrophilic compounds not only boosts the number of absorbed water molecules into the membrane but also increases the possibility of large pores’ formation and water molecules occupation^42^. Therefore, raising the porosity causes the membrane flux to increase^52^. Likewise, increased hydrophilicity in the membrane results in a higher outer surface contact angle, effective in the flux rising^41^. However, the flux decreases with the formation of smaller pores in the membrane^36^. The applied pressure could be another reason for the observed difference in the flux of membranes derived from cigarette butts in this study with the flux of the commercial CA-based membranes. The prepared membranes from CB recycling had a flux of 14.7 LMH and 13.2 LMH under osmotic pressure caused by draw solution containing 1 M sodium chloride, whereas the flux of the CA membrane was reported 15 LMH at a pressure of 100 kPa in the study conducted by Han et al.^42^.

**Table 1:** Characteristics of the prepared membranes.

Membrane	Porosity (%)	Jw (LMH)	Js (gMH)	Js/Jw	Metal rejection (%)		
					Pb	Cr	Cd
Prepared from UCF recycling	61	14.7	3.5	0.24	89.3	91.3	87.6
Prepared from FCB recycling	58	13.2	3.9	0.30	85.2	88.4	85.3

### Removal of heavy metals

The results of prepared membranes’ effectiveness in heavy metals removal are presented in Table 2. At a concentration of 10 mg/L, the removal efficiency of FCB resulting membrane for lead, chromium, and cadmium were 85.2, 88.4, and 85.3%, respectively. In comparison, the removal efficiency of the UCF resulting membrane for these metals at the same concentration were 89.3, 91.3, and 87.6%, respectively. Increasing the concentration of the mentioned metals up to 50 mg/L on average caused the removal efficiency of the FCB and UCF resulting membranes to decrease by 3.53 and 4.06%, respectively. The simultaneous presence of all three metals in the feed solution (10 mg/L of each metal) reduced the removal efficiency of the FCB membranes compared to the concentration of 10 mg/L. However, on average, it was 0.73% better than the removal efficiency with a concentration of 30 mg/L of each metal.

**Table 2:** Removal efficiency of membranes for heavy metals at different concentrations.

Metals	Membrane	Removal efficiency for each metal at different concentrations (%)				Removal efficiency (%) of prepared membrane from FCB at a concentration of 30 mg/L (containing 10 mg/L of Pb and 10 mg/L of Cd and 10 mg/L of Cr)
		10 mg/L	20 mg/L	30 mg/L	50 mg/L	
Pb	UCF	89.3	87.9	86.4	84.7	84.1
	FCB	85.2	84.3	83.1	82.2	
Cd	UCF	87.6	87.1	86.4	83.2	83.9
	FCB	85.3	84.5	83.3	80.7	
Cr	UCF	91.3	90.1	89.2	88.1	86.5
	FCB	88.4	87.1	85.9	85.1	

In previous studies, prepared membranes using polymer at a ratio of 18 wt% were able to remove heavy metals^35,38^. However, in this study, using cigarette butt at a ratio of 15 wt% led to the production of membrane with the ability to remove heavy metal, which is probably due to the effect of the plasticizer in the cigarette filter. In comparison with the performance of the prepared membranes in this study, the efficiency of the CA membrane for nickel removal from the water stream was reported to be more than 93% in a study conducted by Zhao et al.^53^. In 2013, Butler et al. reported that the efficiency of the CA membranes for chromium, lead, copper, and arsenic removal in the forward osmosis process was more than 99%^54^. In 2019, Chen et al. studied the efficiency of CA membranes in lead, chromium, zinc, copper, and mercury removal at a concentration of 100 mg/L, and the mentioned removal rate for metals was more than 99%^55^. Considering that heavy metal removal using the membrane technology depends on physical, chemical, and electrochemical processes as well as hydraulic rules^56,57^, the metal removal efficiency achieved in this study can be explained. Convective transport and the driving force of the draw solution cause heavy metals to transport through the membrane^58^. As the results indicate, the removal efficiency of both membranes for all three studied metals was near 90% because when concentration polarization occurs, the selective character of the membrane can effectively remove heavy metals^59^. The applied low pressure using forward osmosis intensifies the mentioned phenomenon because at low pressure, flux depends on concentration polarization^60^. In this condition, plugging of the pores by smaller particles and the accumulation of large particles on the pores, which can enhance metal removal efficiency, occur slowly. In addition to that, diffusive transport can cause metal ions to move through the membrane regardless of the flow movement^59,61^. In contrast to convective transport, this process depends on the electrochemical properties of the membrane and heavy metal ions as well as polarization concentration^58^. On the other hand, the lack of other ions in used synthetic wastewater can be another reason for not achieving a promising removal efficiency. The presence of other ions can change membrane surface charge by creating repulsive force. It also has an impact on concentration polarization and the tendency of the solution to maintain electro-neutrality on both sides of the membrane. Taking into account these effects, the presence of other metal ions can increase or decrees the removal efficiency^62,63^. Furthermore, as seen in reality, the presence of other compounds in the solution can boost removal efficiency.

### Comparison of the efficiency of the prepared membranes and improvement suggestions

Commercial cellulose acetate can be used to fabricate nanofilters with excellent permeability and high efficiency in reducing salts and ions from the aqueous solution. For example, Su et al. successfully prepared a nanofilter made from cellulose acetate with a permeability of 0.47 LMH and the ability to reduce sodium chloride and magnesium chloride from a synthetic solution by 90 and 96%, respectively. Accordingly, the CA membrane can effectively be used in the forward osmosis process^64^. Moradi Hamedani et al. investigated the efficiency of the CA membranes for the removal of metals such as lead, cadmium, zinc, and nickel. Although an increase in the pressure caused the removal efficiency for all metals to decrease, the CA membrane showed the ability to remove 98% of lead and 70% of other metals^65^. In the study conducted by Idris et al.^66^, the efficiency of a modified CA membrane for lead removal from wastewater was 97.6%^66^. The CA nanofilter used by Figoli et al. to remove cadmium from an aqueous solution showed up to 95% removal efficiency under different pressures and pH values^67^. Yu et al. investigated the ability of the modified cellulose acetate membranes in copper and oil pollution removal from contaminated water. The observed efficiency for copper removal was up to 97% in this study^68^. In another study by Al-Wafi et al. The CA membrane showed an efficiency of 90% for hexavalent chromium removal from an aqueous solution. The researchers successfully increased the removal efficiency to 97% by adding some compounds to the membrane structure^69^. However, mixing the casting solution with additives does not always improve the removal efficiency of the membrane for heavy metals. Nagandaran et al. realized that increasing the ratio of polysulfonate in the casting solution for CA membrane preparation can negatively change the resulting membrane’s pore size, reducing removal efficiency for cadmium, zinc, nickel, and copper ions^51^. Comparing the mentioned removal efficiencies for pure CA membranes and prepared membranes from CB recycling (85 to 90% according to Table 2), it can be said that the removal efficiency of CB-based membranes was acceptable yet less than commercial types. However, this difference can be a result of different operating conditions of the used forward osmosis system and the under-pressure systems used in other studies. Also, the use of the modified membranes by other researchers could be another probable reason.

Considering the better removal efficiency of the pure CA membranes and after reviewing the experiences of other researchers in this field, the following solutions could be presented to improve the CB-based membrane efficiency. Some chemicals such as silver can be used as additives in the membrane preparing process to improve the performance of the resulting membrane. This additive can positively affect the membrane structure and increase its efficiency in reducing pollutants such as microorganisms^70^. Besides, making some changes to the membrane preparation process can improve the structural properties and performance of the resulting membrane. For example, in a study by Nguyen et al., it was stated that the annealing process during fabrication of the CA membranes improves the efficiency of the membranes due to the removal of additives and remained solvents^71^. Mohammadi and Seljuqi examined the effect of preparation conditions on the CA membrane structure and concluded that increasing the polyethylene glycol concentration and water bath temperature during the membrane preparing process improves the thermal resistance of the resulting membrane. Furthermore, it became clear that increasing the polyethylene glycol concentration causes porosity to increase, while porosity decreases with raising the concentration of cellulose acetate and decreasing the water bath temperature^72^. The addition of some chemicals in the membrane preparing process can also improve the membrane structure and performance. For example, Vara et al. successfully reduced the membrane pore size from 15 to less than 2 μm by adding alumina to the casting solution^73^. However, the application of additives can also have some negative side effects. For instance, in the study by Abedini et al., adding titanium oxide led to an increase in thickness and thermal tolerance of the membrane. Meanwhile, the pore size and permeability of the membrane increased, resulting in the removal efficiency reduction^46^. Therefore, the chemicals used for membrane modification and their ratio in the mixture must be chosen carefully in order to achieve the best result. As an example, we can mention the interesting results reported by Nazimuddin et al. They found that the addition of carbon nanotubes as an additive to the casting solution increased the porosity of resulting membrane, but also raised salt rejection to 96%, and the best ratio of polymer to solvent was reported 25 to 75^74^.

### Comparison of the resulting membranes with other cigarette butt recycling products

**Figure 4:**
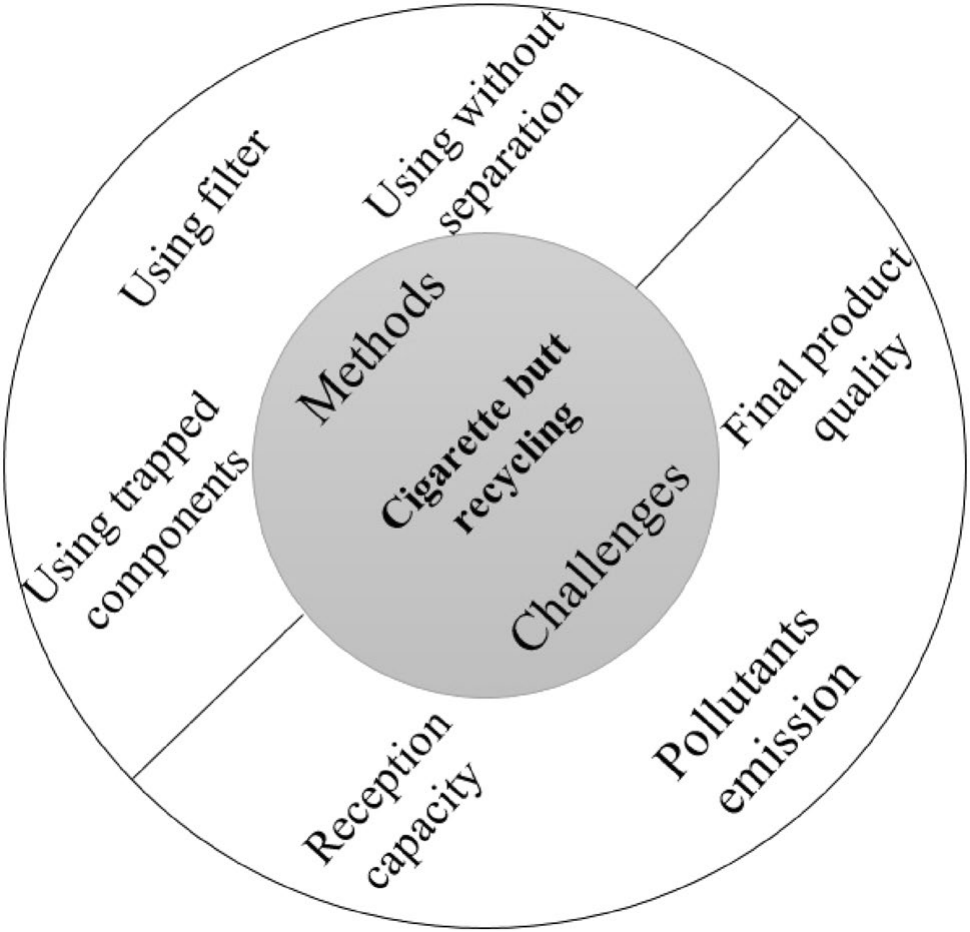
Methods and challenges on CB recycling.

Although the number of products recycled from CBs has increased in recent years, as shown in Fig. 4, there are serious challenges related to this hazardous waste recycling process, hampering the large-scale recycling of CBs^19^.

**Figure 5:**
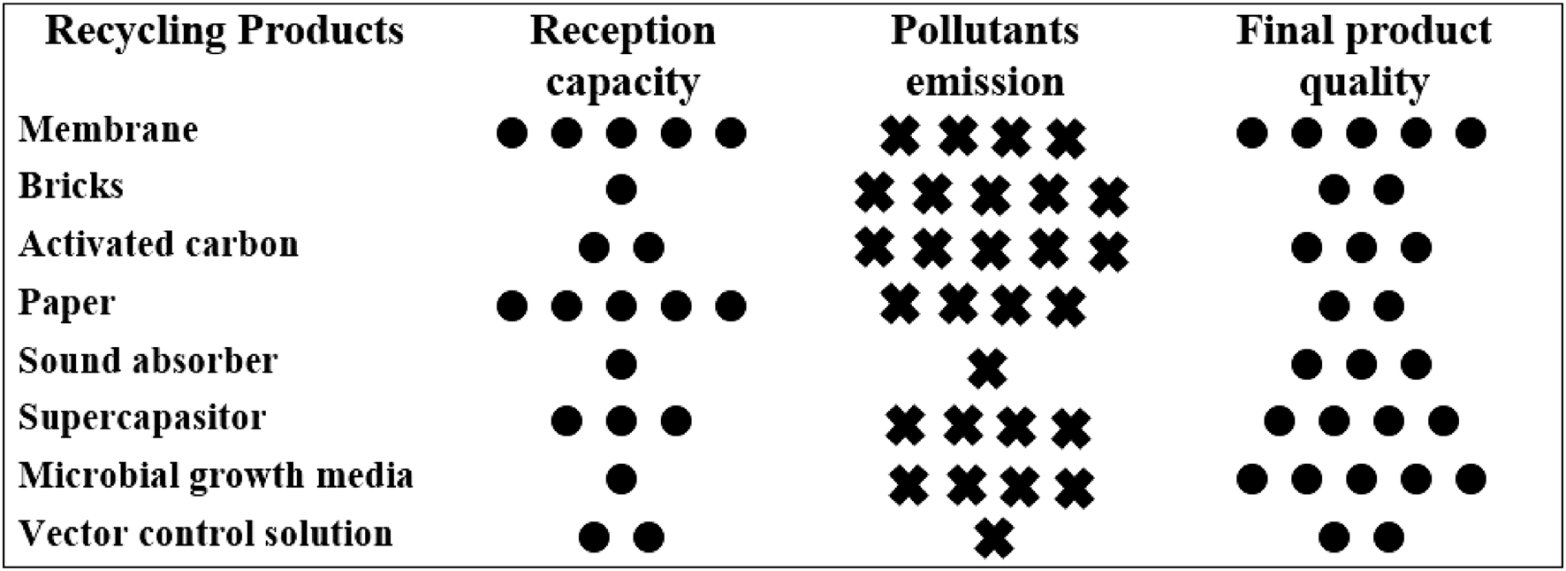
Characteristics of products from CB recycling.

The characteristics of the prepared membrane in this study are compared with other products converted from CB recycling in Fig. 5. One of the most important challenges of CB recycling is the leakage of pollutants in the processing stage in the form of wastewater or airborne pollutants. Considering that cigarette filters are designed to trap pollutants from cigarette smoke, CB waste contains a wide range of pollutants, including heavy metals and toxins^3,6^. These pollutants leak during CB processing, such as washing^19^ and heating^25^. Therefore, converting CBs into products that require fewer processing steps and pollutant leakage is more desirable in terms of environmental consequences. Some CB recycling methods focus on the extraction of trapped chemicals and toxins in the filter, making them more environmentally friendly compared to other CB recycling methods. Membrane production from CB recycling led to the generation of wastewater with various pollutants in the washing steps using water and solvent. From this aspect, the presented method is similar to the CB recycling methods for the production of sound absorbers and super-capacitors. However, since the heat process was not used for CB preparation in this study, there is no threat about the emission of airborne pollutants in contrast to the production of carbon absorbers.

The quality of the final product is a key point to be considered in CB recycling. The presented study results showed that the prepared membrane from CBs had a proper morphology, and its flux and reverse salt rate were similar to the ranges reported for commercial CA membranes. Also, the removal efficiency of the prepared membrane for heavy metals was more than 85%, while the figure was more than 99% for pure CA membranes^54,55^. However, the efficiency of the prepared membrane can be improved by adding specific compounds to the raw material or changing the membrane production process; hence, the quality of the presented product can be considered satisfying.

It is estimated that the annual production of CBs will reach 1.8 million tons by 2025. As a result, the required quantity of CBs in the recycling method plays an important role in managing this prevalent hazardous waste. The recycled product from CBs must be wieldy used and requires the highest number of CBs during the recycling process. In this study, the production of the CA membrane as a widely-used product in various industries was investigated. Since the required cellulose acetate for membrane production came from CB recycling, this product is an excellent option for CB waste management, while this is not the case for some other CBs recycled products. Mohajerani et al. reported that the best ratio of CBs used in brick raw materials is 1% by weight^25^. Therefore, brick production cannot recycle a large amount of CB waste. Furthermore, in the study by Sabzali et al., although the quality of microbial growth substrate medium prepared from obtained from CBs was promising in the study by Sabzali et al. reported to be appropriate and the whole substrate was supplied through CB recycling^22^, but the consumption of these substrates this medium is not widely used in the wastewater industry and does not have the potential to play a considerable role in the world is not enough to make a significant share of manage dealing with 180 several million tons of CBs waste produced annually worldwide. produced worldwide through recycling.

## Conclusion

Membrane production from CB recycling was investigated in this study. According to the results, the produced membrane using the phase inversion technique had macrovoids and a uniform surface structure. The flux in the prepared membrane from CBs was 13.2 LMH, and the reverse salt rate was 3.9 gMH. The comparison of this membrane with the prepared membrane from the unsmoked cigarette filters showed that smoking and processing did not have a serious negative effect on the quality of the final product. The flux and reverse salt in the prepared membrane from unsmoked cigarette filters were 14.7 LMH and 3.5 gMH, respectively. The removal efficiencies of the prepared membrane from CB recycling for lead, chromium, and cadmium were 85.2, 88.4, and 85.3%, respectively, which were on average 3.3% lower than those of the prepared membrane from the unsmoked cigarette filters. Considering the appropriate morphology and heavy metal removal efficiency of the prepared membrane from CB recycling, this product can be an effective solution to tackle CBs waste issue. Besides, another considerable advantage of this recycled product is the fact that the required CA for membrane production completely comes from CB recycling, making the CA membrane an ideal product to manage CBs as the most common litter in the world.

## Data Availability

The datasets generated and analysed during the current study available from the corresponding author on reasonable request.
